# Outcome of emergency patients transported by ambulance during the COVID-19 pandemic in Osaka Prefecture, Japan: a population-based descriptive study

**DOI:** 10.3389/fpubh.2023.1322236

**Published:** 2024-01-11

**Authors:** Yusuke Katayama, Kenta Tanaka, Hisaya Domi, Jun Masui, Shunichiro Nakao, Jotaro Tachino, Tomoya Hirose, Tetsuhisa Kitamura, Jun Oda, Tetsuya Matsuoka

**Affiliations:** ^1^The Working Group to Analyze the Emergency Medical Care System in Osaka Prefecture, Osaka, Japan; ^2^Department of Traumatology and Acute Critical Medicine, Osaka University Graduate School of Medicine, Suita, Japan; ^3^Department of Social and Environmental Medicine, Division of Environmental Medicine and Population Sciences, Osaka University Graduate School of Medicine, Suita, Japan; ^4^Osaka Prefectural Government, Osaka, Japan; ^5^Department of Emergency Medicine, Tane General Hospital, Osaka, Japan; ^6^Rinku General Medical Center, Izumisano, Japan

**Keywords:** COVID-19, pandemic, emergency medicine, EMS system, public health, epidemiology

## Abstract

**Background:**

The novel corona virus (COVID-19) pandemic occurred worldwide. Although an excessive burden was placed on emergency medical institutions treating urgent and severe patients, its impact on patient outcome remains unknown. This study aimed to assess the impact of the COVID-19 pandemic in 2021 on the emergency medical services (EMS) system and patient outcomes in Osaka Prefecture, Japan.

**Methods:**

This was a retrospective descriptive study with a study period from January 1, 2019 to December 31, 2021. We included patients who were transported by ambulance and had cleaned data that was recorded in the ORION system. The study endpoints were the number of patients transported by ambulance and the number of deaths among these patients in each month. To assess the impact of the COVID-19 pandemic on the EMS system, the incidence rate ratio (IRR) and 95% confidence interval (CI) were calculated using 2019 as the reference year. Mortalities were evaluated based on deaths in the emergency department and deaths at 21 days after hospitalization.

**Results:**

The numbers of patients transported by ambulance were 500,194 in 2019, 443,321 in 2020 (IRR: 0.88, 95% CI: 0.87–0.88), and 448,054 in 2021 (IRR: 0.90, 95% CI: 0.89–0.90). In 2019, the number of patients transported by ambulance and who died in the emergency departments was 4,980, compared to 5,485 in 2020 (IRR: 1.10, 95% CI; 1.06–1.44) and 5,925 in 2021 (IRR: 1.19, 95% CI: 1.15–1.24). In 2019, the number of patients who died within 21 days after hospitalization was 11,931, compared to 11,913 in 2020 (IRR; 1.00, 95% CI; 0.98–1.03) and 13,376 in 2021 (IRR; 1.12, 95% CI; 1.09–1.15).

**Conclusion:**

The COVID-19 pandemic decreased the number of ambulance requests and worsened mortality of patients transported by ambulance in Osaka Prefecture during 2021.

## Introduction

Novel coronavirus (COVID-19), confirmed in Wuhan, China in December 2019, spread not only in China but throughout the world (1–7). In Japan, the number of COVID-19 patients exceeded 1.7 million as of December 31, 2021 (8). COVID-19 is characterized by symptoms common to ordinary upper respiratory tract infections, such as fever, cough, general malaise, and some patients are even asymptomatic (2, 5, 7, 9). However, the severely ill patients, which account for about 20% of the patients with COVID-19, require intensive care, mainly mechanical ventilation and extracorporeal membrane oxygenation.

As the number of COVID-19 patients increased, especially in the United States and European countries, the number of healthcare workers infected with COVID-19 also increased, and the healthcare system, including emergency medical services (EMS) and intensive care, experienced a critical situation. The healthcare system in Japan is operated from public healthcare insurance, and the EMS system, through which an ambulance can be called, is a public service (10). Since the outbreak of COVID-19, patients with fever have visited specific medical institutions that can treat COVID-19 and have been diagnosed and treated for COVID-19. However, on weekends including holidays and at night when these medical institutions are not open, patients with sudden fever call for an ambulance and are transported to those emergency medical institutions that provide COVID-19 care. Many of these medical institutions include critical care centers that treat severe trauma and out-of-hospital cardiac arrest (OHCA). As a result, an excessive burden was placed on these emergency medical institutions that treat urgent and severe patients, but the impact of this situation on patient outcomes remains unknown.

Osaka Prefecture is the largest metropolitan area in western Japan, with a population of 8.8 million people and approximately a half million calls for ambulances each year (11). Since the first patient with COVID-19 occurred in Osaka Prefecture on January 23, 2020, the cumulative number of COVID-19 patients in Osaka Prefecture as of December 31, 2021 was 203,790 (12). We previously showed the impact of the spread of COVID-19 in 2020 on the EMS system and patient outcomes of those transported by ambulance (13, 14). However, in Japan, there was a marked increase in the number of COVID-19 patients in 2021 compared to 2020, which may have had a further impact. Therefore, the purpose pf this study was to assess the impact of the COVID-19 pandemic in 2021 on the EMS system and patient outcomes using population-based emergency patient registry in Osaka, Japan.

## Materials and methods

### Study design and settings

This was a retrospective descriptive study with a study period from January 1, 2019 to December 31, 2021. We included patients in this study who were transported by ambulance and who had cleaned data that was recorded in the ORION system. Therefore, we excluded patients who were not registered in the ORION system or had missing data.

In 2020, 8,837,685 people lived in the 1905 km^2^ area of Osaka Prefecture. Of that population, 4,235,956 people (47.9%) were male and 2,441,984 people (25.4%) were older adults, aged 65 years old or more (10). Because the ORION data is anonymized without specific personal data, such as patient name, date of birth, and address, the requirement of obtaining patients’ informed consent was waived. This study was approved by the Ethics Committee of Osaka University Graduate School of Medicine, Suita, Japan (approval number: 15003). This manuscript was written based on the STROBE statement to assess the reporting of cohort and cross-sectional studies (15).

### EMS system and hospitals in Osaka Prefecture and Japan

The EMS system is basically the same as that used in other areas of Japan. In Osaka Prefecture, EMS systems such as ambulance dispatch systems are operated by each local government, and ambulances are dispatched by calling 1-1-9. In 2021, the EMS system was operated by 26 fire departments (298 ambulances) and 26 fire control stations. In 2018, there were 517 medical institutions (105,994 beds) in Osaka Prefecture (16), of which 288 are emergency medical hospitals including 16 critical care centers that are designated to accept patients with life-threatening emergency diseases such as severe trauma and sepsis. Since the introduction of the ORION system, EMS personnel at the scene select the appropriate hospital for emergency patients rather than a dispatcher.

### The ORION system

Information on the system configuration of ORION was previously described in detail (17, 18). The EMS personnel at the scene operate the ORION smartphone app for each emergency patient. All of the data input into the cellphone app, such as vital signs and the time of the call to the hospital for acceptance, are also recorded. The cellphone app data are accumulated in the ORION cloud server, and in cooperation with the dispatched EMS personnel, data managers at each fire department directly input or upload the ambulance record of each emergency patient so that it can be connected with the app data. Furthermore, the operators of each hospital also directly input or upload the patient’s data, such as diagnoses and outcomes, after hospital acceptance. The results of the aggregated data in the ORION system are fed back to every fire department and emergency hospital. The Department of Public Health of Osaka Prefecture can also analyze the effects of health policy on the emergency medical system using these collected data. The ORION system has been in place in all fire departments and emergency hospitals in Osaka Prefecture since January 2016.

### Data collection and quality control

The ORION system checks for errors in the inputted in-hospital data, and the staff of each emergency hospital can correct them if necessary. Through these tasks, cellphone app data, ambulance records, and the in-hospital data such as diagnosis and prognosis can be comprehensively registered for each patient transported by an ambulance. The registered data is cleaned by the Working Group to analyze the emergency medical care system in Osaka Prefecture (17). Among the collected and cleaned data, we excluded inconsistent data that did not contain all of the cellphone app data, ambulance records, and in-hospital data such as diagnosis and prognosis. In addition, we also excluded patients whose sex as registered by the fire department did not match that registered by the hospital or whose sex was missing. We also excluded patients whose age input by the fire department and that by the hospital differed by 3 years or more. When this difference was present, we defined the age input by the hospital as the patient’s true age.

### Endpoints

The primary endpoints of this study were the number of patients transported by ambulance and the number of deaths among these patients for each month. These endpoints were calculated using the ORION dataset. In addition, the principal diagnoses of the patients who died were classified according to the ICD-10.

### Statistical analysis

Firstly, we revealed patient characteristics as descriptive study. Categorical variables were described by real numbers and percentages, while continuous variables were described by medians and interquartile range (IQR). Age groups were categorized as children (0–14 years), adults (15–64 years), and older adults (65 years and older). The reasons for the ambulance call were divided into “fire accident,” “natural disaster,” “water accident,” “traffic accident involving car, ship, or aircraft,” “injury, poisoning, and disease due to industrial accident,” “disease and injury due to sports,” “other injury,” “trauma due to assault,” “acute disease,” “interhospital transport” and “other.”

Secondary, to assess the impact of the COVID-19 pandemic on the EMS system, the incidence rate ratio (IRR) and 95% confidence interval (CI) were calculated using 2019 as the reference year. Next, the number of the dead among these patients by reason for the ambulance call for each month of the above years was calculated, and the IRR and 95% CI were calculated in the same way. The IRR was calculated based on the population of Osaka Prefecture determined by the census in 2020 (11). Mortalities were evaluated based on deaths in the emergency department and deaths at 21 days after hospitalization.

In addition, IRRs and 95% CIs were calculated for subgroup analysis limited to patients who called for an ambulance because of “acute disease.” The age groups were classified as children (0–19 years), adults (20–64 years), and older adults (65 years and older). As in the main analysis, the number of the dead among these patients was calculated on a monthly basis, and the IRR and 95% CI were calculated in the same way. Finally, the odds ratios (ORs) and 95% CIs were calculated to evaluate the percentage of mortality by reason for ambulance call. Statistical analyses were implemented using STATA version 16.0MP (STAT Corp., College Station, TX, United States).

## Results

**Figure 1:**
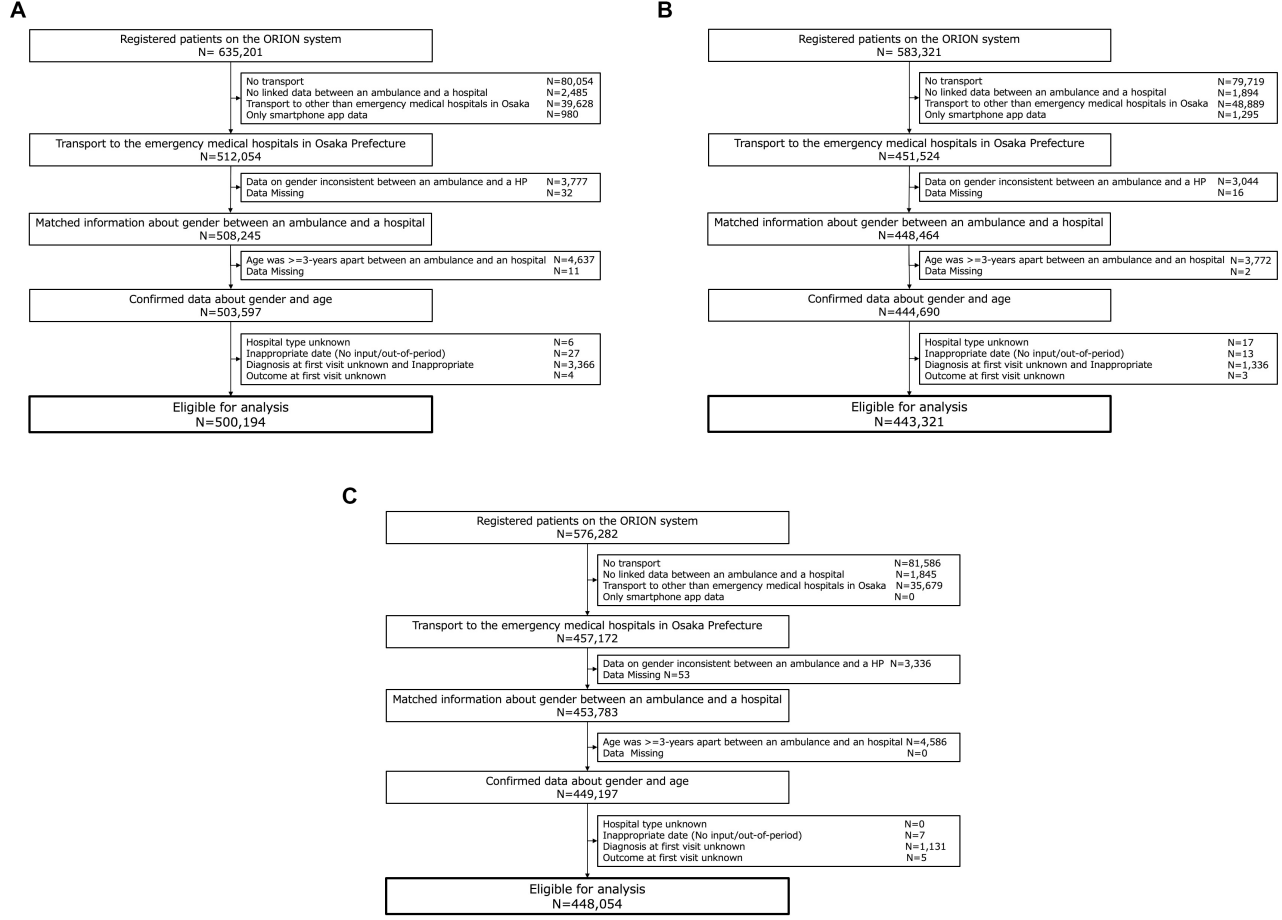
Patient flow in this study.

**Figure 2:**
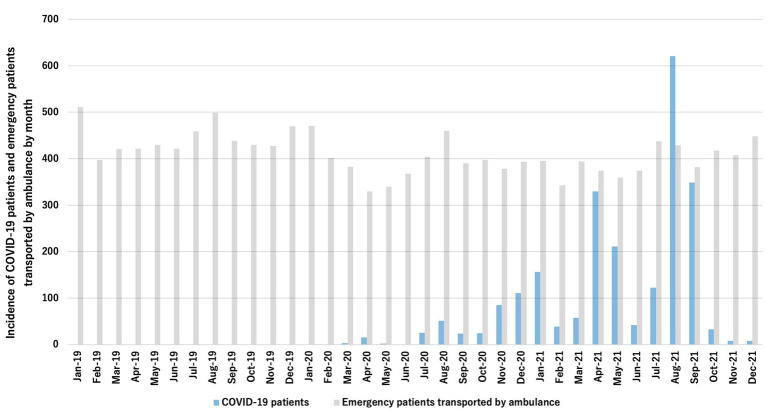
Incidence of emrgency patients transported by ambulance and COVID-19 patients per 100,000 residents.

In this study, we included 1,381,581 data-cleaned patients who registered in the ORION system. Of these patients, 500,206 patients were in 2019, 443,321 patients were in 2020, and 448,054 patients were in 2021 (Figure 1). Figure 2 shows the incidence of COVID-19 patients in Osaka and the incidence of patients transported by ambulance during study periods.

Table 1 shows the patient characteristics in this study. The median of age was 71 years [interquartile range (IQR): 46–82], and 705,972 patients (50.7%) were male. The most common reason for ambulance call was “acute disease” in 946, 778 patients (68.0%), followed by “other injury” (220,149, 15.8%) and “traffic accident” (98,583, 7.1%). The outcome of these patients at the emergency departments were hospitalization in 594,090 (42.7%), discharge home in 760,145 (54.6%), interhospital transfer in 20,840 (1.5%), and death in 16,390 (1.2%). Among the hospitalized patients, the outcomes at 21 days after hospitalization were 167,883 (28.4%) for continuation of hospitalization, 342,102(57.8%) for discharge home, 44,643 (7.5%) for interhospital transfer, and 37,270 (6.3%) for death.

**Table 1:** Clinical characteristics of patients in this study.

Characteristic	Total (*n* = 1,391,581)		2019 (*n* = 500,206)		2020 (*n* = 443,321)		2021 (*n* = 448,054)	
Age, years, median (IQR)	71	(46–82)	70	(43–81)	71	(47–82)	72	(47–83)
**Age group, *n* (%)**								
0–19 years old	94,208	(6.8)	39,592	(7.9)	25,819	(5.8)	28,797	(6.4)
20–64 years old	4,79,463	(34.5)	1,74,002	(34.8)	1,51,924	(34.3)	1,53,537	(34.3)
Over 65 years old	8,17,910	(58.8)	2,86,612	(57.3)	2,65,578	(59.9)	2,65,720	(59.3)
Male, *n* (%)	7,05,972	(50.7)	2,52,828	(50.5)	2,25,222	(50.8)	2,27,922	(50.9)
**Reason for ambulance call, *n* (%)**								
Fire accident	1,095	(0.1)	412	(0.1)	353	(0.1)	330	(0.1)
Natural disaster	47	(0.0)	10	(0.0)	13	(0.0)	24	(0.0)
Water accident	149	(0.0)	52	(0.0)	43	(0.0)	54	(0.0)
Traffic accidents involving car, ship, and aircraft	98,583	(7.1)	36,199	(7.2)	31,134	(7.0)	31,250	(7.1)
Injury, poisoning, and disease due to industrial accident	12,677	(0.9)	4,798	(1.0)	3,933	(0.9)	3,946	(0.9)
Disease and injury due to sport	6,374	(0.5)	2,825	(0.6)	1,604	(0.4)	1,945	(0.5)
Other injury	2,20,149	(15.8)	77,819	(15.6)	71,762	(16.2)	70,568	(15.8)
Trauma due to assault	7,408	(0.5)	2,796	(0.6)	2,474	(0.6)	2,138	(0.5)
Self-induced injury	9,030	(0.6)	2,954	(0.6)	3,067	(0.7)	3,009	(0.6)
Acute disease	9,46,778	(68.0)	3,40,665	(68.1)	3,00,502	(67.8)	3,05,611	(68.0)
Interhospital transfer	88,935	(6.4)	31,497	(6.3)	28,334	(6.4)	29,104	(6.4)
Others	356	(0.0)	179	(0.0)	102	(0.0)	75	(0.0)
**Place, *n* (%)**								
Home	8,33,230	(59.9)	2,93,704	(58.7)	2,67,834	(60.4)	2,71,742	(60.6)
Public space	3,26,861	(23.5)	1,20,642	(24.1)	1,01,342	(22.9)	1,04,877	(23.4)
Workplace	31,956	(2.3)	11,603	(2.3)	10,166	(2.3)	10,187	(2.3)
Road	1,84,581	(13.3)	68,710	(13.7)	59,339	(13.4)	56,532	(12.6)
Other	14,903	(1.1)	5,547	(1.1)	4,640	(1.0)	4,716	(1.1)
**Outcome at emergency department, *n* (%)**								
Hospitalization	5,94,090	(42.7)	2,03,894	(40.8)	1,93,060	(43.5)	1,97,136	(44.0)
Discharge to home	7,60,145	(54.6)	2,84,183	(56.8)	2,38,026	(53.7)	2,37,936	(53.1)
Interhospital transfer	20,840	(1.5)	7,105	(1.4)	6,721	(1.5)	7,014	(1.6)
Death	16,390	(1.2)	4,980	(1.0)	5,485	(1.2)	5,925	(1.3)
Other	116	(0.0)	44	(0.0)	29	(0.0)	43	(0.0)
**Outcomes at 21 days after hospitalization, *n* (%)**								
Continuation of hospitalization	1,67,883	(28.4)	56,489	(27.9)	55,256	(28.7)	56,138	(28.5)
Discharge to home	3,42,102	(57.8)	1,21,131	(59.8)	1,10,606	(57.5)	1,10,365	(56.0)
Interhospital transfer	44,643	(7.5)	12,885	(6.4)	14,675	(7.6)	17,083	(8.7)
Death	37,270	(6.3)	11,931	(5.9)	11,963	(6.2)	13,376	(6.8)

IQR; interquartile range.

Table 2 shows the number of patients transported by ambulance in 2019, 2020, and 2021 by reason for ambulance call and IRRs and 95% CIs. The numbers of patients transported by ambulance were 500,194 in 2019, 443,321 in 2020 (IRR: 0.88, 95% CI: 0.87–0.88), and 448,054 in 2021 (IRR: 0.90, 95% CI: 0.89–0.90). The most common reason for an ambulance call in 2020, 2021, and 2019 was “acute disease,” with the following numbers: 340,655 in 2019, 300,502 in 2020, and 305,611 in 2021. The lowest IRR during the study period for reason for ambulance call was for “disease and injury due to sport” in both 2020 (IRR: 0.57, 95% CI: 0.53–0.60) and 2021 (IRR: 0.69, 95% CI: 0.65–0.73). In terms of the IRR for number of patients transported by ambulance by month, April had the lowest IRR in 2020 (IRR: 0.78, 95% CI: 0.76–0.79), and January had the lowest IRR in 2021 (IRR: 0.78, 95% CI: 0.77–0.79).

**Table 2:** Number of emergency patients registered in the Osaka emergency information research intelligent operation network system.

Reason for ambulance call	January	February	March	April	May	June	July	August	September	October	November	December	Total
**Fire accident**													
2019	58	37	40	34	33	21	38	26	35	29	25	36	412
2020	52	37	28	22	29	18	24	31	12	26	26	48	353
2021	34	28	31	33	23	19	24	22	17	23	35	41	330
IRR: 2019 vs. 2020 (95% CI)	0.90 (0.60–1.33)	1.00 (0.62–1.62)	0.70 (0.42–1.16)	0.65 (0.36–1.14)	0.88 (0.51–1.49)	0.86 (0.43–1.69)	0.63 (0.36–1.08)	1.19 (0.69–2.09)	0.34 (0.16–0.68)	0.90 (0.51–1.58)	1.04 (0.58–1.88)	1.33 (0.85–2.11)	0.86 (0.74–0.99)
IRR: 2019 vs. 2021 (95% CI)	0.59 (0.37–0.91)	0.76 (0.45–1.27)	0.78 (0.47–1.27)	0.97 (0.58–1.62)	0.70 (0.39–1.22)	0.90 (0.46–1.77)	0.63 (0.36–1.08)	0.85 (0.46–1.55)	0.49 (0.26–0.89)	0.79 (0.44–1.42)	1.40 (0.81–2.44)	1.14 (0.71–1.83)	0.80 (0.69–0.93)
**Natural disaster**													
2019	0	0	0	0	0	3	2	1	0	4	0	0	10
2020	8	0	0	0	0	1	2	0	0	2	0	0	13
2021	0	0	0	0	0	0	0	22	0	0	0	2	24
IRR: 2019 vs. 2020 (95% CI)	NA	NA	NA	NA	NA	0.33 (0.01–4.15)	1.00 (0.07–13.80)	NA	NA	0.50 (0.05–3.49)	NA	NA	1.30 (0.53–3.31)
IRR: 2019 vs. 2021 (95% CI)	NA	NA	NA	NA	NA	NA	NA	22.00 (3.56–907.95)	NA	NA	NA	NA	2.40 (1.11–5.62)
**Water accident**													
2019	5	3	6	2	2	2	7	9	9	3	1	3	52
2020	3	4	2	6	3	5	4	2	4	5	2	3	43
2021	3	3	5	2	2	5	9	7	2	6	5	5	54
IRR: 2019 vs. 2020 (95% CI)	0.60 (0.09–3.08)	1.33 (0.23–9.10)	0.33 (0.03–1.86)	3.00 (0.54–30.39)	1.50 (0.17–17.96)	2.50 (0.41–26.25)	0.57 (0.12–2.25)	0.22 (0.02–1.07)	0.44 (0.10–1.59)	1.67 (0.32–10.73)	2.00 (0.10–117.99)	1.00 (0.13–7.47)	0.83 (0.54–1.26)
IRR: 2019 vs. 2021 (95% CI)	0.60 (0.09–3.08)	1.00 (0.13–7.47)	0.83 (0.20–3.28)	1.00 (0.07–13.80)	1.00 (0.07–13.80)	2.50 (0.41–26.25)	1.29 (0.43–4.06)	0.78 (0.25–2.35)	0.22 (0.02–1.07)	2.00 (0.43–12.36)	5.00 (0.56–236.49)	1.67 (0.32–10.73)	1.04 (0.70–1.55)
**Traffic accident involving car, ship, or aircraft**													
2019	2,620	2,510	2,997	3,248	3,024	2,878	3,198	3,068	3,067	3,207	3,223	3,159	36,199
2020	2,635	2,578	2,679	1,891	2,127	2,658	2,843	2,695	2,678	2,820	2,712	2,818	31,134
2021	2,379	2,303	2,590	2,442	2,219	2,625	2,814	2,505	2,432	2,952	2,812	3,177	31,250
IRR: 2019 vs. 2020 (95% CI)	1.01 (0.95–1.06)	1.03 (0.97–1.09)	0.89 (0.85–0.94)	0.58 (0.55–0.62)	0.70 (0.67–0.74)	0.92 (0.88–0.97)	0.89 (0.84–0.94)	0.88 (0.83–0.93)	0.87 (0.83–0.92)	0.88 (0.84–0.93)	0.84 (0.80–0.89)	0.89 (0.85–0.94)	0.86 (0.85–0.87)
IRR: 2019 vs. 2021 (95% CI)	0.91 (0.86–0.96)	0.92 (0.87–0.97)	0.86 (0.82–0.91)	0.75 (0.71–0.79)	0.73 (0.69–0.78)	0.91 (0.86–0.96)	0.88 (0.84–0.93)	0.82 (0.77–0.86)	0.79 (0.75–0.84)	0.92 (0.88–0.97)	0.87 (0.83–0.92)	1.01 (0.96–1.06)	0.86 (0.85–0.88)
**Injury, poisoning, and disease due to industrial accident**													
2019	348	321	370	365	374	385	497	542	455	406	370	365	4,798
2020	279	317	274	282	253	349	344	504	342	368	316	305	3,933
2021	281	257	334	278	259	348	394	354	314	376	384	367	3,946
IRR: 2019 vs. 2020 (95% CI)	0.80 (0.68–0.94)	0.99 (0.84–1.16)	0.74 (0.63–0.87)	0.77 (0.66–0.90)	0.68 (0.57–0.80)	0.91 (0.78–1.05)	0.69 (0.60–0.80)	0.93 (0.82–1.05)	0.75 (0.65–0.87)	0.91 (0.78–1.05)	0.85 (0.73–1.00)	0.84 (0.72–0.98)	0.82 (0.79–0.86)
IRR: 2019 vs. 2021 (95% CI)	0.81 (0.69–0.95)	0.80 (0.68–0.95)	0.90 (0.78–1.05)	0.76 (0.65–0.89)	0.69 (0.59–0.81)	0.90 (0.78–1.05)	0.79 (0.69–0.91)	0.65 (0.57–0.75)	0.69 (0.60–0.80)	0.93 (0.80–1.07)	1.04 (0.90–1.20)	1.01 (0.87–1.17)	0.82 (0.79–0.86)
**Disease and injury due to sport**													
2019	135	166	232	232	252	281	289	295	309	227	213	194	2,825
2020	141	144	51	23	17	76	146	282	225	192	194	113	1,604
2021	71	109	154	137	89	157	276	199	140	222	210	181	1,945
IRR: 2019 vs. 2020 (95% CI)	1.04 (0.82–1.33)	0.87 (0.69–1.09)	0.22 (0.16–0.30)	0.10 (0.06–0.15)	0.07 (0.04–0.11)	0.27 (0.21–0.35)	0.51 (0.41–0.62)	0.96 (0.81–1.13)	0.73 (0.61–0.87)	0.85 (0.69–1.03)	0.91 (0.75–1.11)	0.58 (0.46–0.74)	0.57 (0.53–0.60)
IRR: 2019 vs. 2021 (95% CI)	0.53 (0.39–0.71)	0.66 (0.51–0.84)	0.66 (0.54–0.82)	0.59 (0.47–0.73)	0.35 (0.27–0.45)	0.56 (0.46–0.68)	0.96 (0.81–1.13)	0.67 (0.56–0.81)	0.45 (0.37–0.55)	0.98 (0.81–1.18)	0.99 (0.81–1.20)	0.93 (0.76–1.15)	0.69 (0.65–0.73)
**Other injury**													
2019	7,116	5,753	6,317	6,400	6,157	5,891	6,312	6,518	6,253	6,800	6,785	7,516	77,818
2020	6,936	6,151	5,925	5,021	5,237	5,536	6,037	5,837	5,752	6,645	6,133	6,552	71,762
2021	6,299	5,344	6,116	5,368	5,035	5,066	5,834	5,437	5,129	6,548	6,740	7,652	70,568
IRR: 2019 vs. 2020 (95% CI)	0.97 (0.94–1.01)	1.07 (1.03–1.11)	0.94 (0.91–0.97)	0.78 (0.76–0.81)	0.85 (0.82–0.88)	0.94 (0.91–0.98)	0.96 (0.92–0.99)	0.90 (0.86–0.93)	0.92 (0.89–0.95)	0.98 (0.94–1.01)	0.90 (0.87–0.94)	0.87 (0.84–0.90)	0.92 (0.91–0.93)
IRR: 2019 vs. 2021 (95% CI)	0.89 (0.86–0.92)	0.93 (0.89–0.96)	0.97 (0.93–1.00)	0.84 (0.81–0.87)	0.82 (0.79–0.85)	0.86 (0.83–0.89)	0.92 (0.89–0.96)	0.83 (0.80–0.86)	0.82 (0.79–0.85)	0.96 (0.91–1.00)	0.99 (0.96–1.03)	1.02 (0.99–1.05)	0.91 (0.90–0.92)
**Trauma due to assault**													
2019	268	207	232	232	224	228	226	256	225	217	229	252	2,796
2020	250	225	229	171	197	210	218	185	197	202	185	205	2,474
2021	157	157	193	133	169	165	200	166	147	241	195	215	2,138
IRR: 2019 vs. 2020 (95% CI)	0.93 (0.78–1.11)	1.09 (0.90–1.32)	0.99 (0.82–1.19)	0.74 (0.60–0.90)	0.88 (0.72–1.07)	0.92 (0.76–1.12)	0.96 (0.80–1.17)	0.72 (0.59–0.88)	0.88 (0.72–1.06)	0.93 (0.76–1.13)	0.81 (0.66–0.98)	0.81 (0.67–0.98)	0.88 (0.84–0.93)
IRR: 2019 vs. 2021 (95% CI)	0.59 (0.48–0.72)	0.76 (0.61–0.94)	0.83 (0.68–1.01)	0.57 (0.46–0.71)	0.75 (0.61–0.93)	0.72 (0.59–0.89)	0.88 (0.73–1.08)	0.65 (0.53–0.79)	0.65 (0.53–0.81)	1.11 (0.92–1.34)	0.85 (0.70–1.04)	0.85 (0.71–1.03)	0.76 (0.72–0.81)
**Self-induced injury**													
2019	197	195	245	216	254	291	286	270	254	258	240	247	2,953
2020	265	217	250	184	253	270	315	267	316	297	204	229	3,067
2021	254	259	268	228	224	246	254	248	265	249	239	275	3,009
IRR: 2019 vs. 2020 (95% CI)	1.35 (1.11–1.63)	1.11 (0.91–1.36)	1.02 (0.85–1.22)	0.85 (0.70–1.04)	1.00 (0.83–1.19)	0.93 (0.78–1.10)	1.10 (0.94–1.30)	0.99 (0.83–1.18)	1.24 (1.05–1.47)	1.15 (0.97–1.37)	0.85 (0.70–1.03)	0.93 (0.77–1.11)	1.04 (0.99–1.09)
IRR: 2019 vs. 2021 (95% CI)	1.28 (1.06–1.55)	1.33 (1.10–1.61)	1.09 (0.92–1.31)	1.06 (0.87–1.28)	0.88 (0.73–1.06)	0.85 (0.71–1.01)	0.89 (0.75–1.06)	0.92 (0.77–1.10)	1.04 (0.88–1.24)	0.97 (0.81–1.15)	1.00 (0.83–1.20)	1.11 (0.93–1.33)	1.02 (0.97–1.07)
**Acute disease**													
2019	34,239	25,757	26,544	26,370	27,524	27,131	29,555	32,882	27,935	26,681	26,538	29,499	3,40,655
2020	30,857	25,663	24,224	21,363	21,760	23,247	25,619	30,656	24,781	24,418	23,563	24,351	3,00,502
2021	25,283	21,683	25,002	24,280	23,620	24,286	28,665	28,821	25,163	26,088	25,236	27,484	3,05,611
IRR: 2019 vs. 2020 (95% CI)	0.90 (0.89–0.92)	1.00 (0.98–1.01)	0.91 (0.90–0.93)	0.81 (0.80–0.82)	0.79 (0.78–0.80)	0.86 (0.84–0.87)	0.87 (0.85–0.88)	0.93 (0.92–0.95)	0.89 (0.87–0.90)	0.92 (0.90–0.93)	0.89 (0.87–0.90)	0.83 (0.81–0.84)	0.88 (0.88–0.89)
IRR: 2019 vs. 2021 (95% CI)	0.74 (0.73–0.75)	0.84 (0.83–0.86)	0.94 (0.93–0.96)	0.92 (0.90–0.94)	0.86 (0.84–0.87)	0.90 (0.88–0.91)	0.97 (0.95–0.99)	0.88 (0.86–0.89)	0.90 (0.89–0.92)	0.98 (0.96–0.99)	0.95 (0.93–0.97)	0.93 (0.92–0.95)	0.90 (0.89–0.90)
**Interhospital transport**													
2019	2,897	2,445	2,626	2,732	2,553	2,492	2,662	2,560	2,493	2,581	2,601	2,855	31,497
2020	2,895	2,451	2,367	1,924	1,959	1,996	2,395	2,424	2,282	2,493	2,533	2,615	28,334
2021	2,608	2,180	2,450	2,390	2,323	2,293	2,393	2,542	2,381	2,369	2,460	2,715	29,104
IRR: 2019 vs. 2020 (95% CI)	1.00 (0.95–1.05)	1.00 (0.95–1.06)	0.90 (0.85–0.95)	0.70 (0.66–0.75)	0.77 (0.72–0.81)	0.80 (0.75–0.85)	0.90 (0.85–0.95)	0.95 (0.90–1.00)	0.92 (0.86–0.97)	0.97 (0.91–1.02)	0.97 (0.92–1.03)	0.92 (0.87–0.97)	0.90 (0.89–0.91)
IRR: 2019 vs. 2021 (95% CI)	0.90 (0.85–0.95)	0.89 (0.84–0.94)	0.93 (0.88–0.99)	0.87 (0.83–0.92)	0.91 (0.86–0.96)	0.92 (0.87–0.97)	0.90 (0.85–0.95)	0.99 (0.94–1.05)	0.96 (0.90–1.01)	0.92 (0.87–0.97)	0.95 (0.89–1.00)	0.95 (0.90–1.00)	0.92 (0.91–0.94)
**Other**													
2019	14	9	13	11	13	12	11	7	11	7	11	60	179
2020	9	6	9	11	9	5	8	15	4	11	5	10	102
2021	6	2	5	10	5	12	9	4	8	5	5	4	75
IRR: 2019 vs. 2020 (95% CI)	0.64 (0.25–1.59)	0.67 (0.20–2.10)	0.69 (0.26–1.75)	1.00 (0.39–2.54)	0.69 (0.26–1.75)	0.42 (0.11–1.27)	0.73 (0.25–1.99)	2.14 (0.82–6.21)	0.36 (0.08–1.23)	1.57 (0.56–4.78)	0.45 (0.12–1.42)	0.17 (0.08–0.33)	0.57 (0.44–0.73)
IRR: 2019 vs. 2021 (95% CI)	0.43 (0.13–1.19)	0.22 (0.02–1.07)	0.38 (0.11–1.15)	0.91 (0.35–2.36)	0.38 (0.11–1.15)	1.00 (0.41–2.43)	0.82 (0.30–2.17)	0.57 (0.12–2.25)	0.73 (0.25–1.99)	0.71 (0.18–2.61)	0.45 (0.12–1.42)	0.07 (0.02–0.18)	0.42 (0.32–0.55)
**Total**													
2019	47,897	37,403	39,622	39,842	40,410	39,615	43,083	46,434	41,046	40,420	40,236	44,186	5,00,194
2020	44,330	37,793	36,038	30,898	31,844	34,371	37,955	42,898	36,593	37,479	35,873	37,249	4,43,321
2021	37,375	32,325	37,148	35,301	33,968	35,222	40,872	40,327	35,998	39,079	38,321	42,118	4,48,054
IRR: 2019 vs. 2020 (95% CI)	0.93 (0.91–0.94)	1.01 (1.00–1.03)	0.91 (0.90–0.92)	0.78 (0.76–0.79)	0.79 (0.78–0.80)	0.87 (0.86–0.88)	0.88 (0.87–0.89)	0.92 (0.91–0.94)	0.89 (0.88–0.90)	0.93 (0.91–0.94)	0.89 (0.88–0.90)	0.84 (0.83–0.85)	0.89 (0.88–0.89)
IRR: 2019 vs. 2021 (95% CI)	0.78 (0.77–0.79)	0.86 (0.85–0.88)	0.94 (0.92–0.95)	0.89 (0.87–0.90)	0.84 (0.83–0.85)	0.89 (0.88–0.90)	0.95 (0.94–0.96)	0.87 (0.86–0.88)	0.88 (0.86–0.89)	0.97 (0.95–0.98)	0.95 (0.94–0.97)	0.95 (0.94–0.97)	0.90 (0.89–0.90)

IRR, incidence rate ratio; CI, confidence interval; NA, not assessed. IRR is for 2020 versus 2019.

Table 3 shows the number of patients transported by ambulance, IRRs and 95% CIs by age group for each year. For children, the number of patients transported by ambulance decreased throughout the year in 2020 and 2021, with the lowest IRR in January 2021 (IRR: 0.42, 95% CI: 0.40–0.45). For adults, the IRRs were lowest in April 2020 (IRR: 0.76, 95% CI: 0.74–0.78) and January 2021 (IRR: 0.76, 95% CI: 0.74–0.78). Among older adults, the IRR was lowest in April 2020 (IRR: 0.84, 95% CI: 0.82–0.85), whereas the numbers of patients transported by ambulance in March 2021 (IRR: 0.99, 95% CI: 0.97–1.01), October 2021 (IRR: 1.00, 95% CI: 0.98–1.01), and December 2021 (IRR: 1.01, 95% CI: 0.99–1.03) were similar to those in 2019 before the COVID-19 pandemic.

**Table 3:** Number of emergency patients registered in the ORION system by age group.

Age group	January	February	March	April	May	June	July	August	September	October	November	December	Total
**Total**													
2019	47,897	37,403	39,622	39,842	40,410	39,615	43,083	46,434	41,046	40,420	40,236	44,186	5,00,194
2020	44,330	37,793	36,038	30,898	31,844	34,371	37,955	42,898	36,593	37,479	35,873	37,249	4,43,321
2021	37,375	32,325	37,148	35,301	33,968	35,222	40,872	40,327	35,998	39,079	38,321	42,118	4,48,054
IRR:2019 vs. 2020 (95% CI)	0.93 (0.91–0.94)	1.01 (1.00–1.03)	0.91 (0.90–0.92)	0.78 (0.76–0.79)	0.79 (0.78–0.80)	0.87 (0.86–0.88)	0.88 (0.87–0.89)	0.92 (0.91–0.94)	0.89 (0.88–0.90)	0.93 (0.91–0.94)	0.89 (0.88–0.90)	0.84 (0.83–0.85)	0.89 (0.88–0.89)
IRR:2019 vs. 2021 (95% CI)	0.78 (0.77–0.79)	0.86 (0.85–0.88)	0.94 (0.92–0.95)	0.89 (0.87–0.90)	0.84 (0.83–0.85)	0.89 (0.88–0.90)	0.95 (0.94–0.96)	0.87 (0.86–0.88)	0.88 (0.86–0.89)	0.97 (0.95–0.98)	0.95 (0.94–0.97)	0.95 (0.94–0.97)	0.90 (0.89–0.90)
**Children (age: 0–14 years)**													
2019	4,151	2,784	3,001	3,368	3,481	3,724	3,618	3,254	3,102	2,893	2,766	3,450	39,592
2020	3,328	2,480	2,090	1,748	1,682	1,851	2,173	2,192	2,009	2,213	2,116	1,937	25,819
2021	1,748	1,697	2,262	2,588	2,605	3,036	2,904	2,307	2,026	2,629	2,482	2,513	28,797
IRR:2019 vs. 2020 (95% CI)	0.82 (0.79–0.86)	0.89 (0.85–0.94)	0.70 (0.67–0.74)	0.51 (0.49–0.54)	0.50 (0.48–0.53)	0.56 (0.53–0.58)	0.66 (0.63–0.69)	0.73 (0.70–0.76)	0.69 (0.66–0.72)	0.79 (0.76–0.83)	0.80 (0.76–0.84)	0.60 (0.57–0.63)	0.68 (0.67–0.69)
IRR:2019 vs. 2021 (95% CI)	0.42 (0.40–0.45)	0.61 (0.57–0.65)	0.75 (0.71–0.80)	0.77 (0.73–0.81)	0.75 (0.71–0.79)	0.82 (0.78–0.86)	0.80 (0.76–0.84)	0.71 (0.67–0.75)	0.65 (0.62–0.69)	0.91 (0.86–0.96)	0.90 (0.85–0.95)	0.73 (0.69–0.77)	0.73 (0.72–0.74)
**Adults (age: 15–64 years)**													
2019	14,886	12,338	13,760	13,820	14,200	14,235	15,904	17,296	14,929	14,354	13,411	14,869	1,74,002
2020	14,312	12,370	12,323	10,553	11,145	12,458	14,256	15,734	12,888	12,759	11,719	11,407	1,51,924
2021	11,321	10,300	12,244	11,875	11,687	12,137	15,070	15,973	13,428	13,375	12,635	13,492	1,53,537
IRR:2019 vs. 2020 (95% CI)	0.96 (0.94–0.98)	1.00 (0.98–1.03)	0.90 (0.87–0.92)	0.76 (0.74–0.78)	0.78 (0.77–0.80)	0.88 (0.85–0.90)	0.90 (0.88–0.92)	0.91 (0.89–0.93)	0.86 (0.84–0.88)	0.89 (0.87–0.91)	0.87 (0.85–0.90)	0.77 (0.75–0.79)	0.87 (0.87–0.88)
IRR:2019 vs. 2021 (95% CI)	0.76 (0.74–0.78)	0.83 (0.81–0.86)	0.89 (0.87–0.91)	0.86 (0.84–0.88)	0.82 (0.80–0.84)	0.85 (0.83–0.87)	0.95 (0.93–0.97)	0.92 (0.90–0.94)	0.90 (0.88–0.92)	0.93 (0.91–0.95)	0.94 (0.92–0.97)	0.91 (0.89–0.93)	0.88 (0.88–0.89)
**Older adults (age: ≥65 years)**													
2019	28,864	22,281	22,861	22,654	22,729	21,656	23,561	25,884	23,015	23,173	24,059	25,867	2,86,604
2020	26,690	22,943	21,625	18,597	19,017	20,062	21,526	24,972	21,696	22,507	22,038	23,905	2,65,578
2021	24,306	20,328	22,642	20,838	19,676	20,049	22,898	22,047	20,544	23,075	23,204	26,113	2,65,720
IRR:2019 vs. 2020 (95% CI)	0.92 (0.91–0.94)	1.03 (1.01–1.05)	0.95 (0.93–0.96)	0.82 (0.81–0.84)	0.84 (0.82–0.85)	0.93 (0.91–0.94)	0.91 (0.90–0.93)	0.96 (0.95–0.98)	0.94 (0.93–0.96)	0.97 (0.95–0.99)	0.92 (0.90–0.93)	0.92 (0.91–0.94)	0.93 (0.92–0.93)
IRR:2019 vs. 2021 (95% CI)	0.84 (0.83–0.86)	0.91 (0.90–0.93)	0.99 (0.97–1.01)	0.92 (0.90–0.94)	0.87 (0.85–0.88)	0.93 (0.91–0.94)	0.97 (0.95–0.99)	0.85 (0.84–0.87)	0.89 (0.88–0.91)	1.00 (0.98–1.01)	0.96 (0.95–0.98)	1.01 (0.99–1.03)	0.93 (0.92–0.93)

IRR, incidence rate ratio; CI, confidence interval. IRR is for 2020 versus 2019.

Table 4 shows the number of patients who were transported by ambulance and died in the emergency departments for each year. In 2019, the number was 4,980, compared to 5,485 in 2020 (IRR: 1.10, 95% CI: 1.06–1.44) and 5,925 in 2021 (IRR: 1.19, 95% CI: 1.15–1.24). In 2020, August had the highest IRR (IRR: 1.34, 95% CI: 1.16–1.54) and in 2021, May had the highest IRR (IRR: 1.46, 95% CI: 1.27–1.67).

**Table 4:** Number of deaths in the emergency department after hospital arrival registered in the ORION system.

	January	February	March	April	May	June	July	August	September	October	November	December	Total
2019	664	497	436	399	366	334	320	339	357	350	413	505	4,980
2020	531	519	467	423	412	332	367	453	401	414	507	659	5,485
2021	687	539	484	505	533	364	395	443	389	436	500	650	5,925
IRR (95% CI); 2019 vs. 2020	0.80 (0.71–0.90)	1.04 (0.92–1.18)	1.07 (0.94–1.22)	1.06 (0.92–1.22)	1.13 (0.98–1.30)	0.99 (0.85–1.16)	1.15 (0.98–1.34)	1.34 (1.16–1.54)	1.12 (0.97–1.30)	1.18 (1.02–1.37)	1.23 (1.08–1.40)	1.30 (1.16–1.47)	1.10 (1.06–1.14)
IRR (95% CI); 2019 vs. 2021	1.03 (0.93–1.15)	1.08 (0.96–1.23)	1.11 (0.97–1.27)	1.27 (1.11–1.45)	1.46 (1.27–1.67)	1.09 (0.94–12.7)	1.23 (1.06–1.43)	1.31 (1.13–1.51)	1.09 (0.94–1.26)	1.25 (1.08–1.44)	1.21 (1.06–1.38)	1.29 (1.14–1.45)	1.19 (1.15–1.24)

IRR, incidence rate ratio; CI, confidence interval.

Table 5 shows the number of patients who died within 21 days after hospitalization and the IRR for each year. In 2019, the number was 11,931, compared to 11,963 in 2020 (IRR: 1.00, 95% CI: 0.98–1.03) and 13,376 in 2021 (IRR: 1.12, 95% CI: 1.09–1.15). In the analysis by month, the number of dead patients did not increase or decrease in 2020, whereas in 2021, the number increased in March (IRR: 1.12, 95% CI: 1.03–1.22), April (IRR: 1.37, 95% CI: 1.26–1.49), May (IRR: 1.29, 95% CI: 1.18–1.41), August (IRR: 1.21, 95% CI: 1.11–1.33), and October (IRR: 1.12, 95% CI: 1.02–1.22). There were no reasons for ambulance calls that showed a statically significant impact between 2019 and 2020, and no statistically significant differences were identified between 2019 and 2020.

**Table 5:** Number of deaths among hospitalized patients in the emergency department after hospital arrival registered in the ORION system.

	January	February	March	April	May	June	July	August	September	October	November	December	Total
2019	1,325	1,018	1,006	961	927	808	901	847	890	984	1,096	1,168	11,931
2020	1,251	1,070	1,058	912	898	839	870	915	872	979	1,062	1,237	11,963
2021	1,432	1,011	1,129	1,314	1,195	897	961	1,027	951	1,099	1,101	1,259	13,376
IRR (95% CI); 2019 vs. 2020	0.94 (0.87–1.02)	1.05 (0.96–1.15)	1.05 (0.96–1.15)	0.95 (0.87–1.04)	0.97 (0.88–1.06)	1.04 (0.94–1.15)	0.97 (0.88–1.06)	1.08 (0.98–1.19)	0.98 (0.89–1.08)	0.99 (0.91–1.09)	0.97 (0.89–1.06)	1.06 (0.98–1.15)	1.00 (0.98–1.03)
IRR (95% CI); 2019 vs. 2021	1.08 (1.00–1.17)	0.99 (0.91–1.08)	1.12 (1.03–1.22)	1.37 (1.26–1.49)	1.29 (1.18–1.41)	1.11 (1.01–1.22)	1.07 (0.97–1.17)	1.21 (1.11–1.33)	1.07 (0.97–1.17)	1.12 (1.02–1.22)	1.00 (0.92–1.09)	1.08 (0.99–1.17)	1.12 (1.09–1.15)

IRR, incidence rate ratio; CI, confidence interval.

Table 6 shows the number of deaths in the emergency department by reason for ambulance call. Mortality increased statistically for “acute disease,” from 1.2% (4,166/340,665) in 2019 to 1.5% (4,615/300,502, OR: 1.26, 95% CI: 1.21–1.31) in 2020 and 1.7% (5,049/305,611, OR: 1.36, 95% CI: 1.30–1.41) in 2021. As well, mortality increased statistically for “fire accident,” from 3.9% (16/412) in 2019 to 8.2% (27/330, OR 2.21, 95% CI: 1.12–4.46) in 2021.

**Table 6:** Proportion of deaths in the emergency department registered in the ORION system during the study period.

Reason for ambulance call	Mortality rate % (*n*/*N*)						2020 vs. 2019		2021 vs. 2019	
	2019		2020		2021		OR	(95% CI)	OR	(95% CI)
Fire accident	3.9	(16/412)	4.0	(14/353)	8.2	(27/330)	1.02	(0.45–2.27)	2.21	(1.12–4.46)
Natural disaster	0	(0/10)	0	(0/13)	4.2	(1/24)	NA	—	NA	—
Water accident	38.5	(20/52)	30.2	(13/43)	25.9	(14/54)	0.69	(0.27–1.77)	0.56	(0.22–1.38)
Traffic accident involving car, ship, or aircraft	0.2	(57/36,199)	0.2	(66/31,134)	0.2	(68/31,250)	1.35	(0.93–1.95)	1.38	(0.96–2.00)
Injury, poisoning, and disease due to industrial accident	0.5	(22/4,798)	0.6	(23/3,933)	0.3	(12/3,946)	1.28	(0.68–2.41)	0.66	(0.30–1.40)
Disease and injury due to sport	0	(0/2,825)	0	(0/1,604)	0.1	(2/1,945)	NA	—	NA	—
Other injury	0.4	(340/77,819)	0.5	(345/71,762)	0.5	(373/70,568)	1.10	(0.94–1.28)	1.21	(1.04–1.41)
Trauma due to assault	0.3	(7/2,796)	0.1	(3/2,474)	0.2	(4/2,138)	0.48	(0.08–2.12)	0.75	(0.16–2.94)
Self-induced injury	9.3	(274/2,954)	10.5	(323/3,067)	9.9	(297/3,009)	1.15	(0.97–1.37)	1.07	(0.90–1.28)
Acute disease	1.2	(4,166/340,665)	1.5	(4,615/300,502)	1.7	(5,049/305,611)	1.26	(1.21–1.31)	1.36	(1.30–1.41)
Interhospital transport	0.2	(65/31,497)	0.3	(74/28,334)	0.2	(72/29,104)	1.27	(0.89–1.80)	1.20	(0.85–1.70)
Other	7.3	(13/179)	8.8	(9/102)	8.0	(6/75)	1.24	(0.45–3.26)	1.11	(0.33–3.29)
Total	1.0	(4,980/500,206)	1.2	(5,485/443,321)	1.3	(5,925/448,054)	1.25	(1.20–1.30)	1.33	(1.28–1.38)

OR, odds ratio; CI, confidence interval; NA, not assessed.

Table 7 shows the number of deaths within 21 days after hospitalization by reason for ambulance call. Mortality increased statistically for “acute disease” and “interhospital transport.” In patients with ambulance calls for “acute disease,” the morality rates were 6.9% (9,827/142,147) in 2019, 7.3% (9,856/135,151, OR: 1.06, 95% CI: 1.03–1.09) in 2020, and 7.9% (11,067/139,757, OR: 1.16, 95% CI: 1.13–1.19). In patients with ambulance calls for “interhospital transport,” the morality rates were 4.7% (1,215/25,884) in 2019, 5.4% (1,300/24,102, OR: 1.16, 95% CI: 1.07–1.26) in 2020, and 5.8% (1,398/23,938, OR: 1.21, 95% CI: 1.11–1.31).

**Table 7:** Number of deaths among hospitalized patients registered in the ORION system during the study period.

Reason for ambulance call	Mortality rate % (*n*/*N*)						2020 vs. 2019		2021 vs. 2019	
	2019		2020		2021		OR	(95% CI)	OR	(95% CI)
Fire accident	11.6	(19/164)	6.3	(9/143)	9.7	(13/134)	0.51	(0.20–1.24)	0.82	(0.36–1.83)
Natural disaster	0	(0/3)	0	(0/3)	0	(0/5)	NA	—	NA	—
Water accident	27.8	(5/18)	11.8	(2/17)	32.0	(8/25)	0.35	(0.03–2.65)	1.22	(0.27–5.93)
Traffic accident involving car, ship, or aircraft	2.0	(122/6,221)	1.6	(94/5,705)	2.1	(118/5,500)	0.84	(0.63–1.11)	1.10	(0.84–1.43)
Injury, poisoning, and disease due to industrial accident	1.2	(19/1,536)	1.3	(16/1,270)	1.7	(21/1,239)	1.02	(0.49–2.10)	1.38	(0.70–2.72)
Disease and injury due to sport	0.3	(1/389)	0	(0/233)	0.7	(2/297)	NA	—	2.63	(0.14–155.57)
Other injury	2.4	(583/24,339)	2.2	(533/24,149)	2.5	(597/23,477)	0.92	(0.82–1.04)	1.06	(0.95–1.20)
Trauma due to assault	1.7	(5/289)	1.5	(4/262)	0.5	(1/183)	0.88	(0.17–4.14)	0.31	(0.01–2.83)
Self-induced injury	9.4	(127/1,345)	10.2	(144/1,406)	10.9	(148/1,360)	1.09	(0.84–1.42)	1.17	(0.91–1.52)
Acute disease	6.9	(9,827/142,147)	7.3	(9,856/135,151)	7.9	(11,067/139,757)	1.06	(1.03–1.09)	1.16	(1.13–1.19)
Interhospital transport	4.7	(1,215/25,884)	5.4	(1,300/24,102)	5.8	(1,398/23,938)	1.16	(1.07–1.26)	1.21	(1.11–1.31)
Other	7.9	(8/101)	8.5	(5/59)	6.4	(3/47)	1.08	(0.26–3.95)	0.79	(0.13–3.51)
Total	5.9	(11,931/202,436)	6.2	(11,963/192,500)	6.8	(13,376/196,962)	1.06	(1.03–1.09)	1.16	(1.13–1.19)

OR, odds ratio; CI, confidence interval; NA, not assessed.

## Discussion

This study revealed the outcomes of patients transported by ambulance in Osaka Prefecture from 2019 to 2021. The number of patients transported by ambulance decreased in 2020 and also in 2021 compared to 2019. However, the number of deaths among patients transported by ambulance in 2020 was the same as that in 2019, whereas not only deaths in the emergency department but also deaths among patients transported by ambulances after hospitalization increased in 2021. This population-based descriptive study of the impact of the COVID-19 pandemic will be useful for planning health care systems and policies.

The number of patients transported by ambulance in 2021 was about the same as that in 2020 and was decreased compared to that in the pre-pandemic period. In particular, the number of the patients transported due to traffic accidents and industrial accidents decreased, whereas that of the patients transported due to sports increased slightly. This trend was also observed in other countries (19–26). A study in northwestern Italy reported a decrease in emergency room visits due to the COVID-19 pandemic but an increase in emergency room visits by ambulance (25). A study by Bosson et al. assessing the relationship between hospital admission due to COVID-19 and EMS transports for time-sensitive emergencies in Los Angeles revealed that the number of patients transported by ambulance for traffic accidents decreased during the COVID-19 pandemic, whereas the number of patients transported by ambulance for stroke, ST-segment elevation myocardial infarction, and OHCA increased (24). Thus, the number of these patients may have decreased during the COVID-19 pandemic due to the restriction of socioeconomic activities caused by the lockdown (24). Furthermore, the number of patients transported by ambulance for acute diseases also decreased in 2021 compared to the pre-COVID-19 pandemic period. The impact of the COVID-19 pandemic on changes in patient behavior has been reported in various ways (27, 28). In France, the COVID-19 pandemic reduced initiation of treatment with cardiovascular and antidiabetic drugs (27). In Japan, the COVID-19 pandemic reduced outpatient visits for epilepsy, Parkinson’s disease, and Alzheimer’s disease slightly but significantly in April 2020 (28). This study also found that the COVID-19 pandemic changed patient behavior, such as calling for an ambulance, and that it had not returned to pre-COVID-19 pandemic levels in 2021. The COVID-19 vaccination coverage in Japan was 80% in 2021, and the effect of preventive measures against COVID-19, such as vaccine dissemination, remains clear. We will continue to evaluate changes in patient behavior, such as for ambulance calls, as vaccination coverage increases.

The mortality of patients transported by ambulance in 2020 compared to 2019 did not change, but mortality in 2021 increased. Several studies have reported that patient outcomes were affected by the COVID-19 pandemic (29–31). Surek et al. (29) found that hospitalizations for acute cholecystitis and uncomplicated appendicitis were markedly reduced during the COVID-19 pandemic, whereas hospitalizations for complicated appendicitis and acute mechanical intestinal obstruction increased, as did the mortality from emergency surgery. A study of OHCA in South Korea found that the time from arrival at the scene to the start of resuscitation activities and transport time were prolonged by the need to secure isolation wards and by the increased requirement for personal protective equipment in the prehospital situation (31). In Japan, bystander cardio-pulmonary resuscitation for OHCA patients decreased during the COVID-19 pandemic (30). Thus, factors such as delays in patient access to medical care, decreased treatment performance due to the wearing of infection protection equipment by healthcare workers, and lower rates of prehospital first aid may have affected patient outcomes. These factors were brought about by the need to prevent COVID-19 infection, and widespread use of vaccine may ameliorate these factors. Therefore, we intend to evaluate these effects in the future.

There are several limitations in this study. First, we analyzed IRR on a population basis and did not adjust for various confounding factors. Second, the ORION registry registered patient data from all fire departments and medical institutions only in Osaka Prefecture, so the prognosis of patients transported to medical institutions outside of Osaka Prefecture was not known. Third, we utilized data from a particular region in Japan, which may not be widely applicable elsewhere due to variations in COVID-19 infection rates and insurance systems across different nations. Finally, as this is an observational study, there may be unknown confounding factors.

In conclusion, the COVID-19 pandemic decreased the number of ambulance requests and increased the mortality of patients transported by ambulance in Osaka Prefecture during 2021. The EMS system may have been affected by an increase in special demands, such as the pandemic of infectious diseases.

## Data availability statement

The data analyzed in this study is subject to the following licenses/restrictions: data cannot be shared publicly because of the Protection Ordinance for Personal Information in Osaka Prefecture. Requests to access these datasets should be directed to YK, orion13@hp-emerg.med.osaka-u.ac.jp.

## Author contributions

YK: Conceptualization, Writing – original draft, Data curation, Investigation, Methodology, Project administration, Resources, Visualization, Writing – review & editing. KT: Conceptualization, Data curation, Formal analysis, Methodology, Resources, Software, Visualization, Writing – review & editing. HD: Conceptualization, Methodology, Resources, Writing – review & editing. JM: Conceptualization, Data curation, Methodology, Resources, Writing – review & editing. SN: Conceptualization, Data curation, Resources, Writing – review & editing. JT: Conceptualization, Data curation, Methodology, Resources, Writing – review & editing. TH: Conceptualization, Data curation, Resources, Writing – review & editing. TK: Conceptualization, Data curation, Formal analysis, Methodology, Resources, Software, Validation, Writing – original draft, Writing – review & editing. JO: Conceptualization, Funding acquisition, Methodology, Resources, Supervision, Writing – review & editing. TM: Conceptualization, Data curation, Resources, Supervision, Writing – review & editing.
